# Rapid-onset respiratory failure following diesel aspiration at 4,200 m: a case report

**DOI:** 10.3389/fmed.2026.1802060

**Published:** 2026-04-14

**Authors:** Yan Bai, Xiangyong Yan, Ting Kang, Yan Hou, Xianghui Zhu, Xiaoxiang Liu, Hongning Yang, Fuguo Gao

**Affiliations:** 1 The 940th Hospital of Joint Logistics Support Force of Chinese People's Liberation Army, Lanzhou, China; 2 The 944th Hospital of Joint Logistics Support Force of Chinese People's Liberation Army, Jiuquan, China

**Keywords:** chemical pneumonitis, diesel aspiration, high-altitude illness, occupational exposure, type I respiratory failure

## Abstract

**Background:**

Oral siphoning of diesel fuel carries substantial risks of chemical inhalation injury; however, documented cases of diesel aspiration pneumonia, especially at altitudes >2,500 m, remain rare. The synergistic effects of hydrocarbon toxicity and hypobaric hypoxia in such environments are inadequately characterized.

**Case presentation:**

A 23-year-old man accidentally aspirated approximately 10 mL of diesel while siphoning a fuel line at 4,200 m altitude. Within 5 h, he developed nausea, vomiting, and dry cough, progressing to right-sided pleuritic chest pain, fever (38.2 °C), and hypoxemia (SpO₂ 84%). Arterial blood gas analysis revealed type I respiratory failure (PaO₂ 44.8 mmHg). Chest CT demonstrated patchy consolidations in the right middle lobe and left lower lobe. Laboratory findings included leukocytosis (15.9 × 10⁹/L) and elevated C-reactive protein (72.51 mg/L). Management comprised supplemental oxygen, broad-spectrum antibiotics (meropenem covering anaerobes and Gram-negative bacteria), intravenous methylprednisolone, and nebulized mucolytics.

**Discussion:**

Diesel’s lipophilic and irritant properties disrupt the alveolar-capillary barrier, inciting intense inflammation and pulmonary edema. At high altitude, baseline hypoxemia (SpO₂ 85–90%) synergistically exacerbates ventilation-perfusion mismatch and accelerates respiratory failure. Hypoxia-amplified inflammatory cascades and consequent pulmonary hypertension further increase right ventricular afterload.

**Conclusion:**

Diesel aspiration pneumonia may progress rapidly to life-threatening respiratory failure under hypobaric conditions. Early triple therapy (broad-spectrum antibiotics, systemic corticosteroids, supplemental oxygen) is critical to mitigate complications. This case underscores the imperative for occupational safety interventions prohibiting oral siphoning and heightened vigilance for toxic-hypoxic interactions at high altitude.

## 1 Introduction

Diesel fuel exerts systemic toxicity through inhalation, ingestion, and dermal absorption (1). Although oral siphoning persists as an occupational hazard in specific settings (2), diesel aspiration pneumonia remains clinically underreported, and its pathophysiological evolution at high altitude lacks systematic characterization. At elevations >2,500 m, reduced barometric pressure and inspired oxygen tension profoundly disrupt physiological homeostasis (3), potentiating pulmonary injury. While isolated cases associate diesel aspiration with acute respiratory distress syndrome (ARDS), detailed documentation of bilateral pneumonia secondary to diesel inhalation under high-altitude hypoxia is absent. We describe a 23-year-old man who rapidly developed bilateral pneumonia and respiratory failure after aspirating diesel at 4200 m, aiming to elucidate the synergistic interplay between high-altitude hypoxia and hydrocarbon-induced lung injury.

## 2 Case description

### 2.1 Patient description

The patient was a 23-year-old male of Han ethnicity working as an automotive mechanic. He had no history of chronic conditions such as hypertension, diabetes, or heart disease, and no prior history of infectious diseases including hepatitis or tuberculosis. He reported no smoking or alcohol use, no known food or drug allergies, and no recent surgery, trauma, or family history of illness. Prior to the current presentation, he had been working as a mechanic at an altitude of 4,200 meters.

### 2.2 Case history

During vehicle maintenance, the patient accidentally aspirated approximately 10 mL of diesel fuel while orally siphoning a fuel line. Immediately following aspiration, he developed nausea and vomiting (vomitus consisting of gastric contents), along with a dry cough. Five hours later, he experienced fever with a temperature of 37.6 °C; he rested in bed and did not receive any specific treatment. The following day, he developed sharp right-sided chest pain, and his temperature rose to 38.2 °C. He continued to have no sputum production, dyspnea, or hemoptysis, and subsequently presented to our hospital for further evaluation and management.

### 2.3 Physical examination results

On the day of admission (while breathing room air), vital signs were as follows: temperature 37.8 °C, heart rate 108 beats per minute, respiratory rate 25 breaths per minute, blood pressure 110/56 mmHg, and pulse oxygen saturation (SpO₂) 84%. Physical examination revealed cyanotic lips and localized crackles over the right lung field. The patient was alert and oriented with coherent speech. No jugular venous distension was noted, and the trachea was midline. Cardiac, abdominal, and neurological examinations were unremarkable.

### 2.4 Other investigations

**Figure 1:**
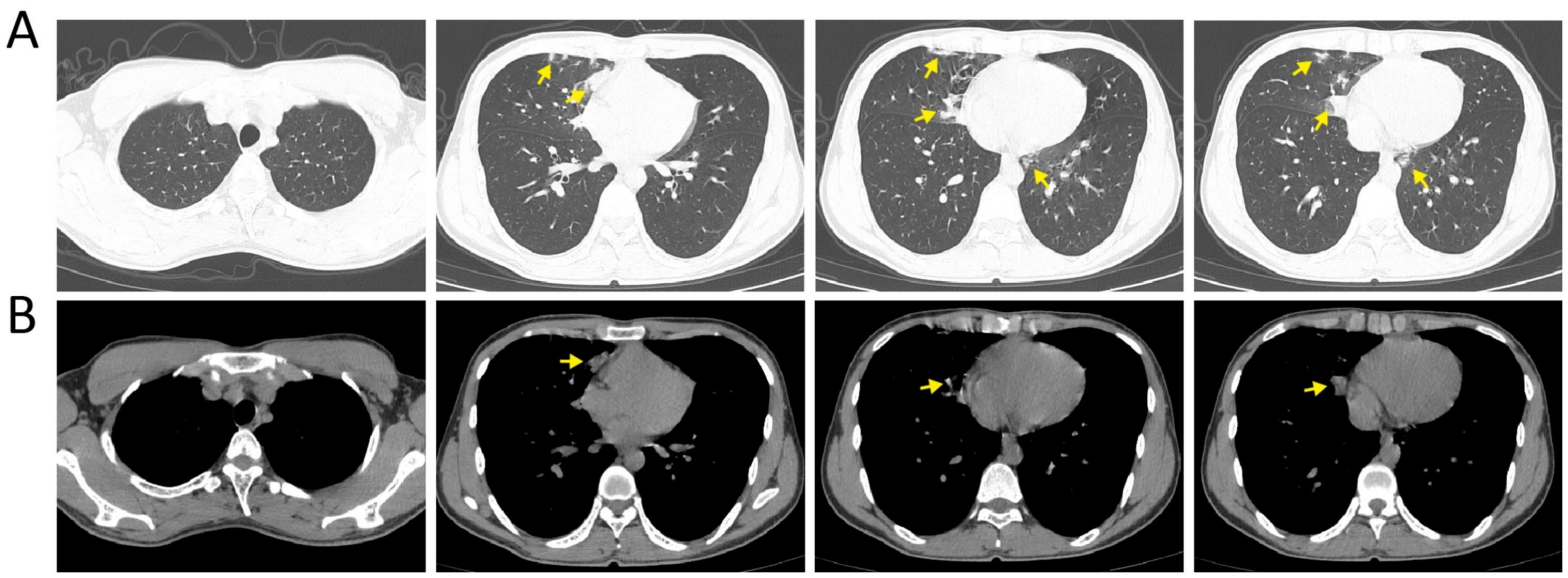
Chest CT on the first day of admission. **(A)** Lung window; **(B)** mediastinal window. Yellow arrows indicate the lesion sites.

Laboratory tests on the day of admission showed: white blood cell count 15.9 × 10⁹/L with neutrophilia (79.8%) and lymphocytopenia (12.8%); red blood cell count 6.41 × 10¹²/L, hemoglobin 202 g/L, platelet count 203 × 10⁹/L; C-reactive protein 72.51 mg/L; coagulation studies revealed mildly elevated fibrinogen (FIB) at 4.62 g/L. Arterial blood gas analysis while breathing room air revealed pH 7.426, PaCO₂ 27.9 mmHg, PaO₂ 44.8 mmHg, bicarbonate (HCO₃^−^) 17.9 mmol/L, and anion gap (AG) 16.2 mmol/L, indicating respiratory alkalosis with concurrent elevated AG metabolic acidosis and hypoxemia. Chest computed tomography (CT) demonstrated patchy areas of increased attenuation in the medial segment of the right middle lobe and the left lower lobe (Figure 1).

### 2.5 Treatment plan

Based on the history of diesel aspiration, imaging findings, and blood-gas derangement. Diagnosis: Chemical pneumonitis secondary to diesel aspiration complicated by acute type I respiratory failure. (with elevated AG metabolic acidosis). Treatment was initiated as follows:

- Supplemental oxygen via face mask (5 L/min).
- Anti-infective therapy: meropenem 1 g intravenously every 8 h (empiric coverage for aerobic and anaerobic bacteria).
- Anti-inflammatory therapy: methylprednisolone sodium succinate 40 mg intravenously once daily (to control excessive inflammatory response).
- Gastroprotective therapy: omeprazole enteric-coated tablets 40 mg orally once daily.
- Mucolytic therapy: bromhexine hydrochloride injection 4 mg intravenously twice daily.
- Nebulized therapy: budesonide suspension 1 mg by nebulization twice daily.

### 2.6 Expected outcome of the treatment plan

The anticipated outcomes of anti-infective, anti-inflammatory, and supportive therapy were to control pulmonary infection, attenuate the inflammatory response, improve oxygenation, correct acid–base disturbances, promote resolution of pulmonary lesions, and ultimately achieve clinical symptom relief, normalization of laboratory parameters, and radiological improvement.

### 2.7 Actual outcome

**Figure 2:**
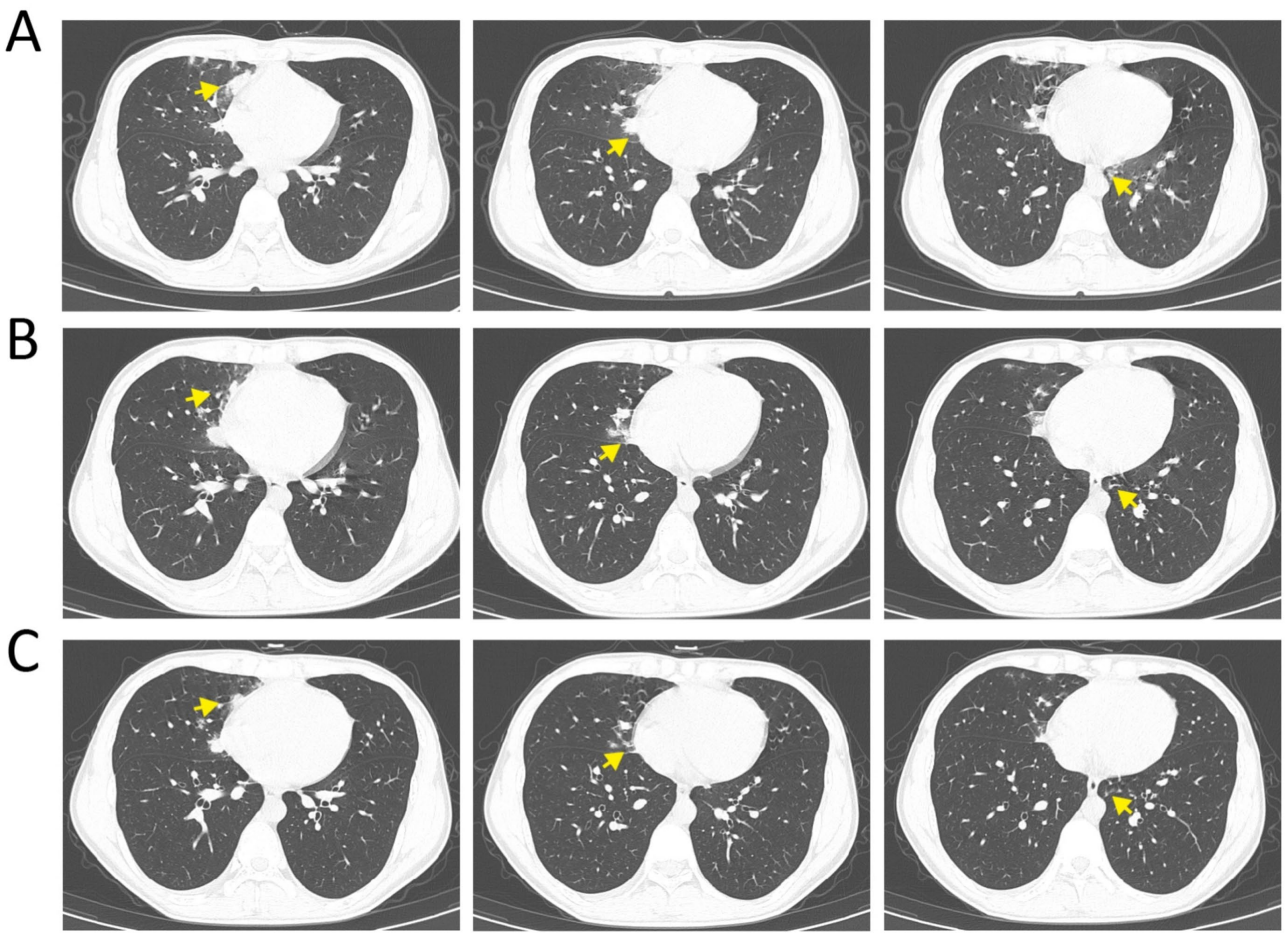
Panels **(A–C)** correspond to chest CT images obtained on hospital days 1, 3, and 7, respectively. Yellow arrows indicate the affected lesions.

On admission, chest CT revealed extensive lung lesions (Figure 2A). On day 3 of treatment, the patient’s body temperature normalized and chest pain resolved. Follow-up laboratory tests revealed: white blood cell count 11.1 × 10⁹/L, neutrophil percentage 69.7%, lymphocyte percentage 23.1%, C-reactive protein 20.68 mg/L, and fibrinogen (FIB) 3.04 g/L. Chest CT showed reduced extent and density of the lesions compared with the initial scan (Figure 2B). By day 7 of treatment, all laboratory parameters had returned to normal, clinical symptoms (fever, chest pain, etc.) had resolved, and chest CT demonstrated further resolution of the lesions (Figure 2C).

## 3 Discussion

Diesel, a complex hydrocarbon mixture predominantly encountered as respirable particulate matter in ambient air, is well-documented to induce airway inflammation, progressive lung function decline, and cardiovascular morbidity (4, 5). Although direct aspiration is uncommon, it characteristically causes chemical inhalation injury (6). Diesel combines potent irritant properties with high lipophilicity (7), rapidly dissolving pulmonary surfactant, disrupting the alveolar-capillary membrane, and inducing necrosis in both type I pneumocytes and capillary endothelial cells. This process triggers an intense inflammatory cascade with robust release of cytokines and lipid mediators, significantly increasing vascular permeability and promoting exudation of plasma proteins into alveolar spaces and interstitium (8). Epithelial denudation, impaired mucociliary clearance, and stagnant secretions create an optimal environment for bacterial colonization, substantially elevating the risk of secondary bacterial pneumonia (9, 10). Systemically absorbed volatile aromatic compounds exert neurotoxic effects, manifesting as headache, confusion, somnolence, seizures, or coma. Massive aspiration may additionally cause mechanical airway obstruction leading to asphyxia (11).

In this case, the patient developed fever within 5 h of aspirating approximately 10 mL of diesel, followed by severe chest pain the next day. This rapidly progressive clinical course is consistent with the mechanism of direct injury to the alveolar–capillary membrane induced by diesel, triggering an acute inflammatory response. Notably, the patient had no sputum production in the early stage, which aligns with the characteristic of chemical injury dominated by exudation and interstitial edema rather than infectious suppuration in the early phase (12). During this stage, exudation and edema are mainly confined to the pulmonary interstitium, with minimal fluid accumulation in the alveolar spaces. Interstitial edema stimulates stretch receptors and vagal nerve endings in the lung tissue, eliciting a neural reflex that manifests as intense and intractable dry cough. Should the injury progress further, interstitial fluid may breach the alveolar epithelium and enter the alveolar spaces, at which point the cough may evolve to produce copious white or pink frothy sputum.

Although definitions of high altitude vary in the literature—some studies consider elevations above 1,500 m as high altitude (13)—at altitudes between 1,500 and 2,500 m, detectable physiological changes may occur, but high-altitude illness is rare. In contrast, when elevations reach 2,500–3,500 m, acute mountain sickness is common during rapid ascent (14). In the field of pulmonary pathophysiology, a threshold of 2,500 m is most commonly adopted to define high altitude (15). The present case occurred at 4200 m, an environment unequivocally associated with hypobaric hypoxia; therefore, we use 2,500 m as the definition of high altitude in this report, which aligns with established frameworks in high-altitude medicine (16, 17). Above this altitude, maladaptive responses to hypoxia typically manifest as headache, dizziness, nausea, fatigue, and sleep disturbances (17); further progression may lead to life-threatening conditions such as high-altitude cerebral edema (18) or pulmonary edema (19). Aspiration pneumonia occurring at such elevations exhibits distinctive pathophysiology, driven by concurrent environmental hypoxia and low barometric pressure. Healthy, unacclimatized individuals at rest exhibit arterial oxygen saturations of 85–90%—significantly lower than the 95–99% observed at sea level (20, 21). Aspiration pneumonia further impairs ventilation-perfusion matching and increases intrapulmonary shunt, compounding hypoxaemia (22). Superimposition of the pneumonia-induced oxygenation deficit upon baseline high-altitude hypoxaemia precipitates rapid progression to profound hypoxaemia and respiratory failure. Hypoxia at altitude also amplifies inflammatory signalling pathways, exacerbating pulmonary inflammation. Pre-existing hypoxic pulmonary vasoconstriction synergizes with pneumonia-induced pulmonary vasoconstriction, increasing right ventricular afterload and potentiating the risk of acute right heart failure (23).

The arterial blood gas analysis in this case showed pH 7.426, PaCO₂ 27.9 mmHg, HCO₃^−^ 17.9 mmol/L, and an AG of 16.2 mmol/L. While breathing room air, the patient’s PaO₂ was only 44.8 mmHg, a value not only below the normal range at sea level but also significantly lower than the expected range for healthy individuals at the same altitude. The blood gas analysis indicated that hyperventilation secondary to hypoxemia induced respiratory alkalosis, which was accompanied by a concurrent high AG metabolic acidosis. The latter was likely primarily attributable to enhanced anaerobic glycolysis and increased lactate production resulting from hypoxemia (24); in addition, infection-related tissue hypoperfusion and metabolic derangements may have contributed. The compensatory decrease in HCO₃^−^ suggests that a respiratory compensatory response to metabolic acidosis had been initiated.

Chemical pneumonitis induced by aspiration of diesel, petrol, gastric acid, or toxic fumes exhibits characteristic imaging patterns (25, 26). Dependent lung regions are predominantly affected: in supine patients, the posterior basal segments of the lower lobes and superior segments of the lower lobes are most commonly involved; in upright patients, the posterior segments of the upper lobes and superior segments of the lower lobes are primarily targeted. The distribution is typically asymmetric or unilateral, with occasional contiguous spread along the bronchial tree. CT typically demonstrates ground-glass opacities (reflecting interstitial edema and inflammatory infiltrates) mixed with consolidations (indicating alveolar filling by exudate, edema, or atelectasis) (27–29).

The imaging findings in this case were consistent with the above description. The patient’s CT revealed lesions located in the medial segment of the right middle lobe and the posterior basal segments of the left lower lobe, with bilateral but asymmetric distribution, consistent with the gravitational deposition pattern of aspirated material. The imaging findings showed mixed ground-glass opacities and consolidations, which represent the characteristic features of concurrent alveolitis and interstitial edema during the acute phase of chemical pneumonitis.

If the initial insult is not halted, secondary infection frequently ensues. Ground-glass and patchy opacities may coalesce into confluent, dense consolidations (30, 31). Anaerobic superinfection can produce cavitation with air-fluid levels. In fulminant or untreated cases, progression to acute respiratory distress syndrome (ARDS) with diffuse “white lung” changes may occur (32). Effective therapy leads to gradual resolution: consolidations decrease in density and extent, ground-glass opacities clear, and late-stage reticular or band-like opacities may emerge, indicating fibrotic repair. The treatment response in this case corroborates this pattern of evolution. Following comprehensive therapy comprising anti-infective treatment with meropenem (covering anaerobes and Gram-negative bacteria), anti-inflammatory therapy with methylprednisolone, continuous facemask oxygen supplementation (5 L/min), and nebulized mucolytic therapy, the patient exhibited marked improvements in both imaging and laboratory parameters by day 3: the white blood cell count decreased from 15.9 × 10⁹/L to 11.1 × 10⁹/L, C-reactive protein decreased from 72.51 mg/L to 20.68 mg/L, and chest CT showed reduction in lesion extent and density. By day 7, all inflammatory markers had normalized completely, and CT demonstrated further resolution of the lesions. Acute-phase management centres on oxygen support. Continuous low-flow or high-flow oxygen—and hyperbaric therapy when available—is essential to maintain adequate oxygenation (33, 34). Fluid resuscitation must be judicious, balancing maintenance of effective circulating volume against the risk of hydrostatic pulmonary oedema. Empirical antibiotics should cover oropharyngeal anaerobes and Gram-negative bacilli; prolonged anti-anaerobic coverage increases the risk of Clostridioides difficile-associated diarrhoea (CDAD) (35).

This first documented case delineates the rapid progression from acute chemical pneumonitis to life-threatening type I respiratory failure following diesel aspiration at 4200 m. The lipophilicity and chemical irritancy of diesel fuel directly disrupted the alveolar–capillary barrier, triggering a vigorous inflammatory exudate manifested clinically as acute pleuritic pain, severe hypoxemia (PaO₂ 44.8 mmHg), and bilateral patchy consolidations on CT. Baseline high-altitude hypoxemia (SpO₂ 85–90%) synergized with pneumonia-induced V/Q mismatch, while hypoxic stress amplified the inflammatory cascade. Early aggressive therapy—including broad-spectrum antibiotics (meropenem, covering anaerobes and gram-negative bacteria), systemic corticosteroids to attenuate inflammation, high-flow oxygen, and meticulous airway management—averted secondary infection and ARDS. This case underscores the unique hazards of diesel aspiration in hypobaric hypoxia and provides a management template for this altitude-related emergency, aiming to improve clinical outcomes.

## Data Availability

The original contributions presented in the study are included in the article/supplementary material, further inquiries can be directed to the corresponding authors.
